# Supplementary material for: Understanding teamwork in rapidly deployed interprofessional teams in intensive and acute care: A systematic review of reviews
Source: PLoS One. 2022 Aug 18;17(8):e0272942. doi: 10.1371/journal.pone.0272942 (PMC9387792; doi:10.1371/journal.pone.0272942)
Supplement: S1 File — This File contains a complete list of the selected articles used in the analysis. (DOCX) [file pone.0272942.s002.docx]

# List of Articles used in study

Almost, J., Wolff, A. C., Stewart‐Pyne, A., McCormick, L. G., Strachan, D., & D’Souza, C. (2016). Managing and mitigating conflict in healthcare teams: an integrative review. *Journal of Advanced Nursing*, *72*(7), 1490–1505. https://doi.org/10.1111/jan.12903

Aufegger, L., Shariq, O., Bicknell, C., Ashrafian, H., & Darzi, A. (2019). Can shared leadership enhance clinical team management? A systematic review. *Leadership in Health Services (Bradford, England)*, *32*(2), 309–335. https://doi.org/10.1108/lhs-06-2018-0033

Baik, D., Blakeney, E. A.-R., Willgerodt, M., Woodard, N., Vogel, M., & Zierler, B. (2018). Examining interprofessional team interventions designed to improve nursing and team outcomes in practice: a descriptive and methodological review. *Journal of Interprofessional Care*, *32*(6), 1–9. https://doi.org/10.1080/13561820.2018.1505714

Barnard, R., Jones, J., & Cruice, M. (2021). Communication between therapists and nurses working in inpatient interprofessional teams: systematic review and meta-ethnography. *Disability and Rehabilitation*, *42*(10), 1–11. https://doi.org/10.1080/09638288.2018.1526335

Buljac-Samardzic, M., Doekhie, K. D., & Wijngaarden, J. D. H. (2020). *Interventions to improve team effectiveness within health care: a systematic review of the past decade*. 1–42. https://doi.org/10.1186/s12960-019-0411-3

Courtenay, M., Nancarrow, S., & Dawson, D. (2013). Interprofessional teamwork in the trauma setting: a scoping review. *Human Resources for Health*, *11*(1), 57. https://doi.org/10.1186/1478-4491-11-57

Dariel, O. P. dit, & Cristofalo, P. (2018). A meta-ethnographic review of interprofessional teamwork in hospitals: what it is and why it doesn’t happen more often. *Journal of Health Services Research & Policy*, *23*(4), 272–279. https://doi.org/10.1177/1355819618788384

Franklin, B. J., Gandhi, T. K., Bates, D. W., Huancahuari, N., Morris, C. A., Pearson, M., Bass, M. B., & Goralnick, E. (2020). Impact of multidisciplinary team huddles on patient safety: a systematic review and proposed taxonomy. *BMJ Quality & Safety*, *29*(10), 1–2. https://doi.org/10.1136/bmjqs-2019-009911

Heip, T., Hecke, A. V., Malfait, S., Biesen, W. V., & Eeckloo, K. (2020). The Effects of Interdisciplinary Bedside Rounds on Patient Centeredness, Quality of Care, and Team Collaboration: A Systematic Review. *Journal of Patient Safety*, *Publish Ahead of Print*. https://doi.org/10.1097/pts.0000000000000695

Husebø, S. E., & Akerjordet, K. (2016). Quantitative systematic review of multi-professional teamwork and leadership training to optimize patient outcomes in acute hospital settings. *Journal of Advanced Nursing*, *72*(12), 2980–3000. https://doi.org/10.1111/jan.13035

Keller, S., Yule, S., Zagarese, V., & Parker, S. H. (2020). Predictors and triggers of incivility within healthcare teams: a systematic review of the literature. *BMJ Open*, *10*(6), e035471. https://doi.org/10.1136/bmjopen-2019-035471

Laurens, N. H., & Dwyer, T. A. (2010). The effect of medical emergency teams on patient outcome: A review of the literature. *International Journal of Nursing Practice*, *16*(6), 533–544. https://doi.org/10.1111/j.1440-172x.2010.01879.x

Lee, H., Ryu, K., Sohn, Y., Kim, J., Suh, G. Y., & Kim, E. (2019). Impact on Patient Outcomes of Pharmacist Participation in Multidisciplinary Critical Care Teams: A Systematic Review and Meta-Analysis*. *Critical Care Medicine*, *47*(9), 1243–1250. https://doi.org/10.1097/ccm.0000000000003830

McNeill, G., & Bryden, D. (2013). Do either early warning systems or emergency response teams improve hospital patient survival? A systematic review. *Resuscitation*, *84*(12), 1652–1667. https://doi.org/10.1016/j.resuscitation.2013.08.006

Noonan, M., Olaussen, A., Mathew, J., Mitra, Smit, D. V., & Fitzgerald, M. (2019). What Is the Clinical Evidence Supporting Trauma Team Training (TTT): A Systematic Review and Meta-Analysis. *Medicina*, *55*(9), 551–14. https://doi.org/10.3390/medicina55090551

Pearson, A., Porritt, K. A., Doran, D., Vincent, L., Craig, D., Tucker, D., Long, L., & Henstridge, V. (2006). A comprehensive systematic review of evidence on the structure, process, characteristics and composition of a nursing team that fosters a healthy work environment. *International Journal of Evidence‐Based Healthcare*, *4*(2), 118–159. https://doi.org/10.1111/j.1479-6988.2006.00039.x

Schmutz, J. B., Meier, L. L., & Manser, T. (2019). How effective is teamwork really? The relationship between teamwork and performance in healthcare teams: a systematic review and meta-analysis. *BMJ Open*, *9*(9), e028280. https://doi.org/10.1136/bmjopen-2018-028280

Welp, A., & Manser, T. (2016). Integrating teamwork, clinician occupational well-being and patient safety – development of a conceptual framework based on a systematic review. *BMC Health Services Research*, *16*(1), 281. https://doi.org/10.1186/s12913-016-1535-y
