# Supplementary material for: Understanding teamwork in rapidly deployed interprofessional teams in intensive and acute care: A systematic review of reviews
Source: PLoS One. 2022 Aug 18;17(8):e0272942. doi: 10.1371/journal.pone.0272942 (PMC9387792; doi:10.1371/journal.pone.0272942)
Supplement: S1 Table — (DOCX) [file pone.0272942.s004.docx]

**Supporting information**

**S1 Table. Overview of Inclusion and exclusion criteria**

| **Inclusion Criteria for title and abstract screening:** |
| --- |
| - paper is a review of peer-reviewed empirical studies (i.e. papers that summarise and analyse primary research); - published in English between 2000 to 2 February 2021 [search date]; - *Any of the following:*   - inter-professional, inter-disciplinary, multi-disciplinary teams in a healthcare setting;   - at least one of intensive emergency, acute and critical care settings;   - discuss teamwork or teams;   - include rapidly convened teams. |
| **Exclusion criteria for full text screening** |
| - non-emergency healthcare settings, e.g., :   - no fit - athletics/ sport;   - no fit - geriatry/ palliative care/ rheumatology/ hospice;   - no fit - not systematic reviews/ case reviews/ book review/ clinical audit;   - no fit - nutrition/ malnutrition/ diabetes/ bariatrics;   - no fit - oncology/ cancer;   - no fit - other healthcare issue;   - no fit - paediatric/ obstetric/ peri, post, neo-natal/ labour/ childcare;   - no fit - pharmacology/ medication;   - no fit - primary healthcare / GP/ dental;   - no fit - psychology/ psychiatric/ mental health;   - no fit – radiology;   - no fit - recovery/ rehabilitation;   - no fit - social care/ community care/ public health;   - no fit - surgery (routine)/ operation;   - no fit - urology/ gynaecology/ gastro-intestinal; - “Cooperation” refers to an organization = e.g., Gulf Cooperation; - Case reviews, clinical standards and primary research e.g. observational studies on defined populations; - reviews that are not summarising primary studies; - Book reviews. |
