# Supplementary material for: Understanding teamwork in rapidly deployed interprofessional teams in intensive and acute care: A systematic review of reviews
Source: PLoS One. 2022 Aug 18;17(8):e0272942. doi: 10.1371/journal.pone.0272942 (PMC9387792; doi:10.1371/journal.pone.0272942)
Supplement: S2 Table — (DOCX) [file pone.0272942.s005.docx]

**Supporting information**

**S2 Table. Contribution of Articles to Themes**

|  | Cohesion and Social Support | Formal Communication | Generic Communication | Inter-personal Relations & Differences | Inter-professional Dynamics | Leadership Influence | Organizational support & culture | Patient outcomes | Professional/ occupational Roles & Demands | Psycho-social traits & Personality | Shared Mental Models | Staff Outcomes | Team Composition, Structure & Proximity |
| --- | --- | --- | --- | --- | --- | --- | --- | --- | --- | --- | --- | --- | --- |
| Almost et al, 2016 | x | x | x | x | x | x | x |  | x | x |  |  | x |
| Aufegger et al, 2019 | x |  | x | x | x | x |  | x | x | x | x | x |  |
| Barnard et al, 2020 | x | x | x | x | x |  | x | x | x |  | x | x | x |
| Baik et al, 2018 |  | x |  |  | x |  |  | x |  |  | x | x | x |
| Buljac-Samardzic et al, 2010 | x |  |  |  |  | x | x |  |  |  |  | x |  |
| Courtenay et al, 2013 | x | x | x |  | x | x | x | x |  | x | x | x | x |
| Franklin et al, 2020 |  | x |  | x |  |  |  | x | x | x |  | x |  |
| Heip et al, 2020 |  | x | x |  | x |  |  | x | x |  | x | x | x |
| Husebø et al, 2016 |  |  | x |  |  | x |  | x |  |  | x |  | x |
| Keller et al, 2020 | x |  |  | x | x | x | x | x | x | x |  |  |  |
| Laurens et al, 2010 |  |  |  |  | x |  | x |  | x |  |  |  | x |
| Lee et al, 2019 |  |  |  |  |  |  |  | x | x |  |  |  | x |
| McNeill et al, 2013 |  |  |  |  |  |  |  | x | x |  |  |  |  |
| Noonan et al, 2019 |  |  | x | x | x |  |  | x |  |  |  |  |  |
| Pearson et al, 2006 | x |  | x | x | x |  | x | x | x |  |  | x | x |
| Petit dit Dariel et al, 2018 | x |  | x | x | x | x | x | x | x |  | x | x | x |
| Schmutz et al, 2019 | x |  |  |  | x | x | x | x | x |  |  | x | x |
| Welp et al, 2016 | x |  |  | x | x | x | x |  | x |  |  | x | x |
