# Supplementary material for: Understanding teamwork in rapidly deployed interprofessional teams in intensive and acute care: A systematic review of reviews
Source: PLoS One. 2022 Aug 18;17(8):e0272942. doi: 10.1371/journal.pone.0272942 (PMC9387792; doi:10.1371/journal.pone.0272942)
Supplement: S3 Table — (DOCX) [file pone.0272942.s006.docx]

**Supporting information**

S3 Table. Types of healthcare teams, occupation of team members, and clinical settings

|  | **Type of healthcare teams** | | | **Team members** | | | **Healthcare settings** | | | |
| --- | --- | --- | --- | --- | --- | --- | --- | --- | --- | --- |
|  | **Mono-professional** | **Inter-disciplinary** | **Inter-professional** | **Nurses** | **Other Health Professionals** | **medical** | **Acute/ Emergency/ Intensive** | **Cardiac/ Surgical** | **Paediatric/ Palliative/ Rehab** | **Educational/ Simulation** |
| Almost et al, 2016 | x |  | x | x | x | x | x |  |  |  |
| Aufegger et al, 2019 |  |  | x |  |  | x | x | x |  |  |
| Barnard et al, 2020 |  |  | x | n/a | n/a | n/a | n/a | n/a | n/a | n/a |
| Baik et al, 2018 |  | x | x | x | x |  | x | x | x | x |
| Buljac-Samardzic et al, 2010 | x | x | x | x | x | x | x |  | x |  |
| Courtenay et al, 2013 |  | x | x | x | x | x | x |  |  |  |
| Franklin et al, 2020 |  | x | x | x | x | x | x |  |  |  |
| Heip et al, 2020 |  | x | x | x | x | x | x | x |  |  |
| Husebø et al, 2016 |  |  | x | x | x | x | x |  | x |  |
| Keller et al, 2020 |  | x | x | x | x | x | x |  |  |  |
| Laurens et al, 2010 |  |  | x | x | x | x | x |  |  | x |
| Lee et al, 2019 |  |  | x | x | x | x | x | x | x |  |
| McNeill et al, 2013 |  | x | x | x | x | x | x | x |  |  |
| Noonan et al, 2019 |  | x | x | x |  | x | x |  |  | x |
| Pearson et al, 2006 |  | x | x | x |  |  | x | x | x |  |
| Petit dit Dariel et al, 2018 |  |  | x | n/a | n/a | n/a | x | n/a | n/a |  |
| Schmutz et al, 2019 | x |  | x | x | x | x | x |  |  | x |
| Welp et al, 2016 | x | x |  | x | x | x | x | x | x |  |
