# Supplementary material for: Understanding teamwork in rapidly deployed interprofessional teams in intensive and acute care: A systematic review of reviews
Source: PLoS One. 2022 Aug 18;17(8):e0272942. doi: 10.1371/journal.pone.0272942 (PMC9387792; doi:10.1371/journal.pone.0272942)
Supplement: Attachment — Submitted filename: Response to Reviewers.docx [file pone.0272942.s007.docx]

July 4th, 2022

Dear Editorial Team,

Thank you very much for reviewing our article entitled “Understanding teamwork in rapidly deployed interprofessional teams in intensive and acute care: a systematic review of reviews” We very much appreciated your kind and constructive feedback.

As per your feedback and suggestions we edited the supporting information, including legends for all the figures and Table 2 has been linked as cross reference in the text. We can also confirm that all the 18 articles used in the review have been included as in line citations in the text, in the bibliography of the manuscript and a list of reviews used has been uploaded as supporting information. Following your instructions, we have uploaded a version of the manuscript with track changes named ‘*Revised Manuscript with Track Changes’* and one without named *‘Manuscript’*.

Further, in relation to point 3 in the edit requests, our submission is a systematic narrative review, and as such we do not have any statistical calculations, however as using a graph modeling software (i.e., Gephi) requires the reformatting of a NVIVO generated Matrix of the shared references between thematic themes, the the values needed to develop the graph, i.e., a) the Matrix of references from NVIVO as well as the corresponding reformatted b) Edgetable and c) Nodetable, have been uploaded as supporting information in the submission files in line with the email correspondence with the editorial team.

Thank you very much again for the opportunity to publish in PLOS One, for your kind review of this manuscript and for the constructive feedback.

On behalf of the research team, sincerely,

Stefan Schilling
